# Association of CYP gene polymorphisms with breast cancer risk and prognostic factors in the Jordanian population

**DOI:** 10.1186/s12881-019-0884-x

**Published:** 2019-09-02

**Authors:** Laith N. AL-Eitan, Doaa M. Rababa’h, Mansour A. Alghamdi, Rame H. Khasawneh

**Affiliations:** 1 Department of Applied Biological Sciences, Jordan University of Science and Technology, Irbid, 22110 Jordan; 2 Department of Biotechnology and Genetic Engineering, Jordan University of Science and Technology, P.O. Box 3030, Irbid, 22110 Jordan; 3 College of Medicine, King Khalid University, Abha, Saudi Arabia; 4 Department of Hematopathology, King Hussein Medical Center (KHMC), Jordan Royal Medical Services (RMS), Amman, 11118 Jordan

**Keywords:** Breast cancer, Prognostic factors, CYP, CYP19A1, CYP1A

## Abstract

**Background:**

Single nucleotide polymorphisms (SNPs) in several CYP genes have been associated with altered breast cancer (BC) risk in different populations. Despite this, there is a dearth of information on the roles of these SNPs in Jordanian BC patients. Therefore, this study aims to determine if there is any single nucleotide polymorphism (SNP) within *CYP19A1*, *CYP2C19, CYP2C9, CYP1B1, CYP3A4,* and *CYP1A2* genes associated with BC in the Jordanian population. In addition, this work investigates the association between selected BC prognostic factors and variants of the aforementioned CYP candidate genes.

**Methods:**

Blood samples were withdrawn from 221 BC patients and 218 healthy volunteers recruited from the Jordanian population. Genomic DNA was withdrawn and, after quantification and quality control, was genotyped using the Sequenom MassARRAY® system (iPLEX GOLD). Statistical analysis was then carried out to assess allelic and genotypic frequencies as well as genetic association between cases and controls.

**Results:**

The *CYP19A1* SNP rs7176005 (*p* < 0.0045) and the *CYP1A2* SNP rs762551 (*p* = 0.004) were significantly associated with BC risk. However, no such association was found for the screened SNPs of the *CYP2C9, CYP1B1, CYP2C19* and *CYP3A4* genes. Regarding the prognostic factors of BC, several of the screened SNPs were associated with different pathological and clinical features.

**Conclusions:**

Certain CYP genes, particularly *CYP19A1* and *CYP1A2*, were associated with BC risk and development in the Jordanian population.

## Background

Several recent studies have focused on identifying breast cancer (BC) susceptibility genes. It has been reported that pathogenic mutations account for up to 10% of worldwide BC cases [1]. The cytochrome P450 (CYPs) genes that encode for hemoproteins and have major functions in drug metabolism, are involved in the majority of metabolic and clearance processes [2]. Certain CYP genes have been implicated in cancer formation and development due to their roles in promoting oxidative stress, activating procarcinogens, and inactivating anticancer drugs [3, 4]. Not all CYP genes have an equal impact on the disease, as inherited genetic variation at the individual and population levels lead to interethnic differences in cancer risk and treatment response [5]. Examples of such genes include *CYP19A1*, *CYP2C19, CYP2C9, CYP1B1, CYP3A4,* and *CYP1A2*, all of which will be investigated over the course of this study.

The *CYP19A1* gene encodes for the enzyme aromatase, the latter of which is targeted in BC therapy by aromatase inhibitors (AI) due to its critical role in estrogen biosynthesis [6]. *CYP19A1* polymorphisms have been found to modulate circulating estrogen levels, alter tumor characteristics, contribute to AI-associated arthralgia, exacerbate AI-associated bone loss, and improve letrozole efficacy in BC patients [7–11]. On a similar note, the *CYP19A* gene encodes for a hepatic enzyme that is most well-known for its metabolism of the antiplatelet drug clopidogrel, as polymorphisms in this gene could lead to fluctuating therapeutic effect [12]. Asian BC patients undergoing tamoxifen therapy were not impacted by *CYP19A* polymorphisms, but the CYP19A*17 polymorphism was associated with decreased BC risk in Caucasians [13, 14]. Furthermore, the *CYP2C9* gene is responsible for a substantial proportion of phase I metabolism, but its highly polymorphic nature gives rise to changes in metabolic activity and potential adverse drug reactions [15]. In tamoxifen-treated BC patients, *CYP2C9* polymorphisms were thought to influence rates of disease-free survival as well as tumor characteristics, but no significant association was found in the Asian population [13, 16].

Although its role in drug metabolism is not as clearly delineated, the overexpression of *CYP1B1* in tumour cells gives credence to its importance in cancer research [17]. In American and Chinese women, *CYP1B1* polymorphisms were not associated with BC risk, but, in combination with other factors, certain *CYP1B1* alleles were related to BC risk in the Finnish-Caucasian and Turkish populations [18–21]. In contrast, the *CYP3A4* gene is heavily involved in the deactivation and biotransformation of one-third of clinically used drugs [22]. In the context of BC, the CYP3A4*1B polymorphism was significantly associated with early onset of puberty, the latter of which increases BC risk [23]. Moreover, the *CYP3A4* gene was found to inactivate the antineoplastic drug docetaxel and potentially compound its side effects [24, 25]. Lastly, the *CYP1A2* gene is known to metabolize a number of procarcinogens as well as anticancer drugs (like tamoxifen), and its activity is influenced by dietary patterns [26]. The *CYP1A2* AA genotype was found to strip the protective effect of caffeine against BC from patients, while patients with the CYP1A2*1F AA genotype experienced slower ER-positive tumor growth upon coffee consumption [27, 28].

The primary aim of the present study was to determine whether any association existed between certain single nucleotide polymorphisms (SNPs) of the *CYP19A1*, *CYP2C19, CYP2C9, CYP1B1, CYP3A4,* and *CYP1A2* genes and BC in the Jordanian population. A secondary objective investigated the association between selected BC prognostic factors and variants of the aforementioned CYP candidate genes.

## Methods

### Study cohort

Female participants were recruited from the Jordanian population at the Jordanian Royal Medical Hospital after obtaining written informed consent. Ethical approval for the present study was obtained from the Institutional Review Board (IRB) at Jordan University of Science and Technology.

A total of 473 blood samples were collected from unrelated Jordanian women including 231 healthy volunteers and 242 were diagnosed with breast cancer. Both the cases and controls were randomly chosen and adjusted to be matched with regard to age, sex, and ethnic origin.

### Extraction of genomic DNA

5 ml of blood were withdrawn from each volunteer and subject to DNA extraction procedures using the DNA Purification KitWizard® Genomic (Promega, USA). The purified DNA underwent quality control testing via agarose gel electrophoresis (to check for integrity) and measured by the Nano-Drop ND-1000 UV-Vis Spectrophotometer (BioDrop, UK).

### Genotyping

Multiplex PCR was used to amplify Loci of candidate SNPs followed by a primer extention process (Mass EXTEND) resulting in allele-specific DNA products. Mass spectrometry was used for minisequencing reaction product analysis. Afterward, the extension PCR products were separated onto a 384 well spectroCHIP and placed into the MALDI-TOF (Matrix Assisted Laser Desorption/Ionization Time-of-Flight) mass spectrometer. Finally, a software system (Spectro TYPER-RT (RT for real-time) was used to analyze the results. SNPs were genotyped using Sequenom iPLEX (Sequenom, San Diego, CA, USA). MassARRAY assay design (version 3.1) software (Sequenom MassARRAY system) was used to design the PCR in addition to the single base extension primers (SBE) at the AGRF; Australian Genome Research Facility (AGRF) (Melbourne, Australia).

### Statistical analysis

The equation for the Hardy-Weinberg equilibrium (HWE), *p*^*2*^ *+ 2pq + q*^*2*^ *= 1*, was used for all SNPs in both groups using the Chi-square (χ^2^) goodness of-fit test.Variations between cases and controls were calculated by employing Pearson’s chi-squared using the Statistical Package for the Social Sciences (SPSS), version 25.0 (SPSS, Inc., Chicago, IL). The odds ratio (OR) was also calculated using binary logistic regression with 95% confidence intervals (CI).On the other hand, genetic association analysis using different genetic models was carried out in this study, *p*-values were considered to be statistically significant only if they were less than 0.05. Haploview program (version 4.2) was used to perform haplotype analysis test for linkage disequilibrium (LD) and to allow for the analysis of LD blocks and haplotype.

### Correction for multiple testing

Multiple comparison method of Li and Ji (2005) was used to estimate the effective number of SNPs (*N*_*em*_) [29], which employs a modification of an earlier approach by Nyholt (2004) [30]. Modified Bonferroni procedure was applied to determine a target alpha level (0.05/ *N*_*em*_) that would maintain an overall significance level of 0.0045 or less.

## Results

### Samples characteristics

General characteristics of the study cohort in the current research was summarized and categorized by AL-Eitan et al., (2017) [31]. All controls were unrelated healthy females from Jordan with approximate average age of 50.8 ± 12.6 years and their ages ranged from 24 to 90 years.

Data obtained for this study was available for 221 female patients who were diagnosed with BC. All participants were unrelated with averages of ages at BC diagnosis (51.1 ± 16.5), at pregnancy (22.6 ± 2), at menarche (13.8 ± 0) and at menopause (48.31 ± 4.5). The estimated average of body mass index (BMI) for patients group was 31.28 ± 3.48. In addition, 66% of patients have practiced breastfeeding while 34% of them did not breastfed at any stage of their life.

Other clinical features were also investigated, co-morbidities, 46% of BC patients suffered from other complications such as hypertension, coronary artery disease, asthma, and diabetes mellitus. Furthermore 32% of patients have family history with BC disease while 27% of the patients had allergy. However, life style such as smoking was included in this analysis, we found that only 30% of the cases were smokers.

In terms of BC pathological features, 80% of all cases had been diagnosed with invasive ductal carcinoma compared to the 20% of patients who found with in-situ ductal carcinoma. With regard to hormone receptor status, estrogen and progesterone receptors were found on the malignant cells of 74 and 44% of patients, respectively, while 40% of patients were positive for (human epidermal growth factor receptor 2) (HER2) expression. Certain pathological features such as nodal involvement, have been investigated, 82% of cases were reported with lymph node involvement while 48.4% of the cases showed axillary lymph node metastatic. In addition, patients were grouped into two categories depending on the differentiation rate; low and mild differentiation (62%) and highly differentiated tumor (38%). likewise, stages of tumor progression for patients were divided into two group grade 1, 2 (PT1 + PT2; 90.3%) and grade 3, 4 (PT3 + PT4; 9.7%). Molecular subtyping of BC depending on estrogen, progesterone, and HER2 status to divide the disease into subtypes: luminal A (ER(+) and /or PR(+) Her2 (−)), luminal B (ER(+) and /or PR(+) Her2 (+)), triple negative(ER(−) and /or PR(−) Her2 (−)). Finally, In this study, 47% of patients were L.A, 41% were L.B while 12% were T.N [32].

### Quality control (QC)

Table 1 illustrates the investigated SNPs and their information, in addition the table shows the genotype call rates that ranged from 96 to 97%. After quality control, 222 patient out of 242 and 218 controls out of 231 were included in the analysis.

**Table 1:** List of genes, their SNPs, positions, and genotyping data based on the whole cohort (*N* = 449)

Gene	SNP_ID	Position^a^	SNP	SNP Location	Assay pass rate^b^
*CYP19A1*	rs7176005	15:51339082	C > T^MA^	2 KB Upstream Variant	97%
	rs6493497	15:51338638	G > A^MA^	2 KB Upstream Variant	96%
	rs700519	15:51215771	G > A^MA^	Missense Variant	97%
	rs10046	15:51210789	G > A^MA^	3 Prime UTR Variant	96%
	rs4646	15:51210647	A^MA^ > C	3 Prime UTR Variant	97%
*CYP2C9*	rs1799853	10:94942290	C > T^MA^	Missense Variant	96%
	rs4086116	10:94947445	C > T^MA^	Intron Variant	96%
*CYP2C19*	rs4244285	10:94781859	G > A^MA^	Synonymous Variant	94%
*CYP1B1*	rs10175368	2:38080719	C > T^MA^	Ningle Nucleotide Variant	96%
*CYP3A4*	rs35599367	7:99768693	G > A^MA^	Intron Variant	97%
*CYP1A2*	rs762551	15:74749576	C^MA^ > A	Intron Variant	97%

*MA* minor allele

^a^ Chromosome positions are based on NCBI Human Genome Assembly Build

^b^ Ratio of the number of discordant genotypes to the number of duplicates

### Minor allelic frequency of the investigated CYP450 candidate gene SNPs

Six CYP450 genes essential to drug metabolism were included in this study. Table 2 displays the investigated SNPs within these genes and the allelic distribution frequency for each gene’s minor allele as well as the HWE *p*-value. In this study one SNP (rs408611) of CYP2C9 did not fulfil HWE and was excluded (*P* = 0.036 < 0.05) from the genetic analysis.

**Table 2:** Minor allele frequencies among breast cancer patients and healthy controls and the HWEc *P*-value of CYP450 candidate gene polymorphisms

Gene	SNP ID	Cases (*n* = 221)			Controls (*n* = 218)		
		MA	MAF^a^	HWE^b^ *p*-value	MA	MAF	HWE *p*-value
*CYP19A1*	rs7176005	T	0.23	0.85	T	0.15	0.18
	rs6493497	A	0.22	0.56	A	0.15	0.18
	rs700519	A	0.03	0.68	A	0.02	0.75
	rs10046	A	0.42	0.27	A	0.44	0.50
	rs4646	A	0.29	0.33	A	0.29	0.25
*CYP2C9*	rs1799853	T	0.14	0.084	T	0.15	0.063
	rs4086116	T	0.22	0.17	T	0.23	0.036
*CYP2C19*	rs4244285	A	0.11	0.74	A	0.08	0.84
*CYP1B1*	rs10175368	T	0.29	0.87	T	0.26	0.16
*CYP3A4*	rs35599367	A	0.02	0.70	A	0.03	0.70
*CYP1A2*	rs762551	C	0.33	0.88	C	0.37	0.10

*MA* minor allele

N/A: not applicable

^a^MAF: minor allele frequency

^b^HWE: Hardy—Weinberg equilibrium

### Association of CYP450 SNPs with breast cancer (BC)

The influence of the selected polymorphisms within the *CYP19A1*, *CYP2C9*, *CYP2C19, CYP1B1, CYP3A4,* and *CYP1A2* genes on BC in Jordanian population was investigated via a genetic association analysis between cases and controls. Table 3 shows the frequency distribution for both the alleles and the genotypes in the cases and controls. For most of the SNPs, the frequencies of the variant alleles were slightly higher in the cases than in the controls. For example, 23% of the cases carried the variant allele T of the *CYP19A1* SNP rs7176005 compared to 15% of the controls.

**Table 3:** Association of the investigated CYP450 candidate gene polymorphisms and breast cancer (BC)

Gene	SNP ID	Allelic and Genotypic Frequencies in Cases and Controls						
		Allele/Genotype	Cases: total number = 222 n(%)	Controls: total number = 218 n(%)	*P*-value*	Pearson Chi-square	Odd Ratio	CI (95%)
*CYP19A1*	rs7176005	C	341(0.77)	370(0.85)	0.002	9.408	0.584	0.4132–0.8254
		T	101(0.23)	64(0.15)				
		CC	132(0.6)	155(0.71)	0.003	11.06	5.3538	1.1497–24.9301
		CT	77(0.35)	60(0.28)				
		TT	12(0.05)	2(0.01)				
	rs6493497	G	343(0.78)	368(0.85)	0.005	7.57	0.615	0.4341–0.8711
		A	97(0.22)	64(0.15)				
		GG	12(0.05)	2(0.01)	0.008	9.627	5.7049	1.2236–26.5984
		GA	73(0.33)	60(0.28)				
		AA	135(0.61)	154(0.71)				
	rs700519	G	430 (0.97)	425(0.98)	0.534	0.385	0.7588	0.3164–1.8196
		A	12 (0.03)	9(0.02)				
		AG	12(0.05)	9(0.04)	0.530	0.394	0.7536	0.3109–1.8266
		GG	209(0.95)	208(0.96)				
	rs10046	G	252(0.58)	243(0.56)	0.537	0.381	1.0883	0.8318–1.424
		A	182(0.42)	191(0.44)				
		AA	34(0.16)	42(0.19)	0.585	1.071	0.6577	0.3764–1.1493
		GA	114(0.53)	107(0.49)				
		GG	69(0.32)	68(0.31)				
	rs4646	C	313(0.71)	309(0.71)	N/A	N/A	0.9972	0.7454–1.3342
		A	129(0.29)	127(0.29)				
		AA	22(0.1)	22(0.1)	0.996	0.008	0.9683	0.4867–1.9264
		CA	85(0.38)	83(0.38)				
		CC	114(0.52)	113(0.52)				
*CYP2C9*	rs1799853	C	377(0.86)	367(0.85)	0.424	0.638	1.1665	0.7991–1.7029
		T	59(0.14)	67(0.15)				
		CC	166(0.76)	159(0.73)	0.752	0.569	0.8187	0.2772–2.4179
		CT	45(0.21)	49(0.23)				
		TT	7(0.03)	9(0.04)				
*CYP2C19*	rs4244285	G	383(0.89)	390(0.92)	0.060	3.515	0.6413	0.4019–1.0233
		A	49(0.11)	32(0.08)				
		AA	3(0.01)	1(0.0)	0.170	3.543	2.175	0.2152–21.9809
		AG	43(0.2)	30(0.14)				
		GG	170(0.79)	180(0.85)				
*CYP1B1*	rs10175368	C	310(0.71)	318(0.74)	0.350	0.87	0.8682	0.6452–1.1684
		T	128(0.29)	114(0.26)				
		CC	109(0.5)	121(0.56)	0.340	2.156	0.7297	0.3512–1.5163
		CT	92(0.42)	76(0.35)				
		TT	18(0.08)	19(0.09)				
*CYP3A4*	*rs35599367*	G	431(0.98)	425(0.97)	0.974	0.001	1.0141	0.435–2.3642
		A	11(0.02)	11(0.03)				
		GA	11(0.05)	11(0.05)	0.974	0.001	1.0145	0.4304–2.3914
		GG	210(0.95)	207(0.95)				
*CYP1A2*	rs762551	A	295(0.67)	274(0.63)	0.225	1.472	1.188	0.8993–1.5695
		C	145(0.33)	160(0.37)				
		AA	98(0.45)	95(0.44)	0.004	10.916	0.3712	0.1968–0.7003
		AC	99(0.45)	84(0.39)				
		CC	23(0.1)	38(0.18)				

*CI* confidence interval

*P-Value < 0.0045 considered as significant

The *CYP19A1* SNPs rs7176005 and rs6493497 were significantly associated with BC in terms of both their alleles and genotypes (*p*-value< 0.05). According to our findings the variant allele (T) of rs7176005 was significantly higher among cases (23%) than it among controls (15%). In similar way, rs6493497 variant allele (A) found more frequent among patients than it within controls. We suggest that the variants alleles of *CYP19A1* SNPs; rs7176005 and rs6493497 could be an influence factors for increasing the breast cancer risk.

In addition, the rs762551 SNP of the CYP1A2 gene was also found to be significantly associated with BC with regard to its genotype (*p*-value = 0.00426207) but not to its allele (*p*-value = 0.2250304). Our results revealed that the variant allele (C) of rs762551 among controls was higher than it among cases. Furthermore, the CC genotype among cases (23%) was less than it among controls (38). However according to our statistical significant result, we propose that the CC genotype of the rs762551 SNP of the CYP1A2 gene may act as protective factor against breast cancer progression and development.

No significant correlation was found between the rest of the screened SNPs of the *CYP2C9, CYP1B1, CYP2C19* and *CYP3A4* genes and breast cancer (*p*-value> 0.05).

Different genetic models have been incorporated into the genetic association analysis in this study. Table 4 summarizes these different models (which included dominant, additive, and recessive genetic models) and shows the chi-squared values differentiating between the cases and controls. The *CYP19A1* SNP rs7176005 was significantly associated with breast cancer for both the Rare Hz (TT) vs Het (CT) (χ^2^ = 4.57) and the Rare Hz (TT) vs Common Hz (CC) (χ^2^ = 8.44) genetic models. Similarly, the *CYP19A1* SNP rs6493497 was also associated with BC for both of the aforementioned genetic models. In addition, a connection between the *CYP1A2* SNP rs762551 and BC was observed for the Rare Hz (CC) vs Het (AC) genetic model (χ^2^ = 4.92). No such link to BC was found for any of the other investigated SNPs using these genetic models.

**Table 4:** Genetic association analysis for the investigated CYP450 candidate gene polymorphisms using different genetic models

Gene	SNP ID	Category Test	Odds Ratio	95% CI	Chi square*
***CYP19A1***	rs7176005	Het (CT) vs Common Hz (CC)	1.51	1–2.27	**3.87**
		Rare Hz (TT) vs Het (CT)	4.68	1.01–21.69	**4.57**
		Rare Hz (TT) vs Common Hz (CC)	7.05	1.55–32.05	**8.44**
	rs6493497	Het (AG) vs Common Hz (GG)	1.39	0.92–2.1	2.43
		Rare Hz (AA) vs Het (AG)	4.93	1.06–22.9	**4.94**
		Rare Hz (AA) vs Common Hz (GG)	6.84	1.5–31.13	**8.13**
	rs700519	Het (AG) vs Common Hz (GG)	1.33	0.55–3.22	0.39
		Rare Hz (AA) vs Het (AG)	NA	NA	NA
		Rare Hz (AA) vs Common Hz (GG)	NA	NA	NA
	rs10046	Het (AG) vs Common Hz (GG)	1.05	0.69–1.61	0.05
		Rare Hz (AA) vs Het (AG)	0.76	0.45–1.28	1.06
		Rare Hz (AA) vs Common Hz (GG)	0.8	0.45–1.4	0.62
	rs4646	Het (AC) vs Common Hz (CC)	1.02	0.68–1.51	0.01
		Rare Hz (AA) vs Het (AC)	0.98	0.5–1.9	0
		Rare Hz (AA) vs Common Hz (CC)	0.99	0.52–1.89	0
***CYP2C9***	rs1799853	Het (CT) vs Common Hz (CC)	0.88	0.56–1.39	0.3
		Rare Hz (TT) vs Het (CT)	0.85	0.29–2.46	0.09
		Rare Hz (TT) vs Common Hz (CC)	0.74	0.27–2.05	0.33
***CYP2C19***	rs4244285	Het (AG) vs Common Hz (GG)	1.52	0.91–2.53	2.58
		Rare Hz (AA) vs Het (AG)	2.09	0.21–21.1	0.41
		Rare Hz (AA) vs Common Hz (GG)	3.18	0.33–30.84	1.11
***CYP1B1***	rs10175368	Het (CT) vs Common Hz (CC)	1.34	0.9–2	2.11
		Rare Hz (TT) vs Het (CT)	0.78	0.38–1.6	0.46
		Rare Hz (TT) vs Common Hz (CC)	1.05	0.53–2.11	0.02
***CYP1A2***	rs762551	Het (AC)vs Common Hz (AA)	1.14	0.76–1.71	0.42
		Rare Hz (CC) vs Het(AC)	0.51	0.28–0.93	**4.92**
		Rare Hz (CC)vs Common Hz(AA)	0.59	0.33–1.06	3.18

*Significant values are indicated in bold (χ^2^ > 3.84 and *p*-value< 0.05)

Adjusted *p*-value (Bonferonni correction) = 0.0045

### Association of CYP450 SNPs with breast cancer (BC) prognostic factors

The prognostic factors of BC can be broadly categorized into clinical and pathological features. Clinical features encompass factors like body mass index, smoking, co-morbidity, and age at first BC diagnosis, while pathological features involve progesterone and estrogen receptor statuses, tumor stage, and lymph node involvement, among others. Table 5 demonstrates the relationship between a number of different clinical and pathological features of BC and the investigated *CYP19A1* SNPs. The *CYP19A1* SNPs rs10046 and rs4646 were found to be significantly associated with age at first BC diagnosis (*p*-value = 0.007) and lymph node involvement (*p*-value = 0.022). In contrast, the *CYP19A1* SNP rs700519 was significantly associated with both the age at menopause (*p*-value = 0.002) and age at menarche (*p*-value = 0.04).

**Table 5:** Association between different *CYP19A1* SNP genotypes and the clinico-pathological features of breast cancer (BC)

Clinical features	*CYP19A1*				
	rs10046	rs7176005	rs700519	rs6493497	rs4646
Body mass index ^b^ n = 221	0.510	0.570	0.811	0.547	0.092
Age at first pregnancy ^b^ *n* = 195	0.122	0.200	0.825	0.200	0.956
Age at BC diagnosis ^b^ *n* = 221	0.007	0.681	0.836	0.789	0.921
Allergy ^a^ n = 221	0.936	0.935	0.401	0.967	0.856
Age at menarche ^b^ n = 221	0.989	0.421	0.044	0.405	0.383
Breastfeeding status ^a^ *n* = 221	0.401	0.095	0.367	0.127	0.721
Age at menopause ^b^ *n* = 108	0 .290	0.736	0 .002	0 .736	0.797
Family history ^a^ n = 221	0.571	0.121	0.927	0.138	0.123
Co-morbidity ^a^ n = 221	0.104	0.557	0.924	0.566	0.905
Smoking ^a^ *n* = 216	0.166	0.607	0.059	0.580	0.617
Pathological features					
Progesterone receptor status ^a^ *n* = 198	0.157	0.754	0.867	0.719	0.755
Estrogen receptor status ^a^ *n* = 191	0.714	0.463	0.159	0.339	0.227
Human epidermal growth factor receptor 2 marker (HER2) ^a^ *n* = 139	0.945	0.445	0.493	0.485	0.468
Heteromolecular BC markers^a^ *n* = 138	0.510	0.788	0.915	0.797	0.754
Tumor differentiation ^a^ *n* = 197	0.273	0.851	0.918	0.860	0.906
Axillary lymph nodes ^a^ n = 221	0.844	0.793	0.584	0.687	0.297
Tumor stage ^a^ *n* = 208	0.491	0.118	0.922	0.080	0.236
Histology classification ^a^ *n* = 209	0.722	0.475	0.407	0.507	0.320
Tumor size ^b^ n = 208	0.726	0.547	0.879	0.472	0.448
Lymph node involvement ^a^ n = 221	0.953	0.681	0.617	0.691	0.022

^a^ Pearson’s chi-squared test was used to determine genotype-phenotype association

^b^ Analysis of variance (ANOVA) test was used to determine genotype-phenotype association

*P*-Value < 0.0045 considered as significant

Table 6 shows the relationship between the same BC prognostic factors and the *CYP2C9*, *CYP1A2*, *CYP3A4* and *CYP2C19* SNPs. None of the clinical or pathological features of BC were associated with the *CYP2C9* SNPs. However, the rs762551 of the *CYP1A2* gene was found to be significantly associated with age at menopause (*p*-value = 0.034), Human epidermal growth factor receptor 2 marker (HER2) (*p*-value = 0.028), histology classification (*p*-value = 0.011), and lymph involvement (*p*-value = 0.001) but not with any of the clinical features. Likewise, the *CYP3A4* SNP rs35599367 was significantly associated with age at first pregnancy (*p*-value = 0.009) and tumor stage (*p*-value = 0.04). Lastly, the *CYP2C19* SNP rs4244285 was significantly correlated with HER2 (*p*-value = 0.02).

**Table 6:** Association between different *CYP2C9, CYP1A2, CYP3A4* and *CYP2C19* SNP genotypes and the clinico-pathological features of breast cancer (BC)

Clinical features	*CYP2C9*	*CYP1A2*	*CYP1B1*	*CYP3A4*	*CYP2C19*
	rs1799853	rs762551	rs10175368	rs35599367	rs4244285
Body mass index ^b^ n = 221	0.078	0.178	0.552	0.286	0.834
Age at first pregnancy ^b^ n = 195	0.144	0.292	0.179	0.032	0.785
Age at BC diagnosis ^b^ n = 221	0.511	0.675	0.295	0.391	0.132
Allergy ^a^ n = 221	0.721	0.927	0.859	0.146	0.920
Age at menarche ^b^ n = 221	0.834	0.738	0.905	0.869	0.931
Breastfeeding status ^a^ n = 221	0.250	0.450	0.196	0.009	0.161
Age at menopause ^b^ n = 108	0 .356	0 .034	0 .554	0 .161	0 .266
Family history ^a^ n = 221	0.562	0.167	0.521	0.494	0.294
Co-morbidity ^a^ n = 221	0.659	0.456	0.774	0.822	0.349
Smoking ^a^ n = 216	0.305	0.705	0.899	0.341	0.528
Pathological features					
Progesterone receptor status ^a^ n = 198	0.213	0.118	0.378	0.554	0.213
Estrogen receptor status ^a^ n = 191	0.409	0.208	0.286	0.511	0.409
Human epidermal growth factor receptor 2 marker (HER2) ^a^ n = 139	0.495	0.028	0.109	0.566	0.495
Heteromolecular BC markers^a^ n = 138	0.691	0.043	0.024	0.914	0.081
Tumor differentiation ^a^ n = 197	0.285	0.577	0.498	0.734	0.285
Axillary lymph nodes ^a^ n = 221	0.956	0.587	0.179	0.346	0.956
Tumor stage ^a^ n = 208	0.743	0.153	0.469	0.048	0.743
Histology classification ^a^ n = 209	0.708	0.011	0.238	0.118	0.708
Tumor size ^b^ *n* = 208	0.407	0.433	0.991	0.318	0.407
Lymph node involvement ^a^ n = 221	0.194	0.001	0.406	0.516	0.194

^a^ Pearson’s chi-squared test was used to determine genotype-phenotype association

^b^ Analysis of variance (ANOVA) test was used to determine genotype-phenotype association

*P*-Value < 0.0045 considered as significant

The heteromolecular BC markers PR, ER, and HER2 were also investigated to their recently elucidated prognostic and predictive roles in the disease. BC can be divided into three classifications based on marker expression: Luminal A (ER(+) and /or PR(+) and HER2-neu (−)), Luminal B (ER(+) and /or PR(+) and HER2-neu(+)), and Triple Negative (ER(−), PR(−) and HER2-neu (−)). (Tables 5 and 6) show the relationship between each of these BC classifications and the investigated SNPs. The *CYP1B1* SNP rs10175368 and the *CYP1A2* SNP rs762551 both showed an association with the different BC classifications, with *p*-values of 0.024 and 0.043, respectively.

### Haplotypic analysis

Haplotype was further studied as a part of the genetic association analysis, our findings revealed a strong linkage disequilibrium between five *CYP19A1* SNPs (rs10046, rs4646, rs6493497, rs700519 and rs7176005) that formed one block. However, no significant association was found between any of these haplotypes and BC in Jordanian Arabs (Table 7).

**Table 7:** Association between breast cancer (BC) and different haplotypes

Haplotype CYP19A1 Block (rs10046, rs4646, rs6493497,rs700519 and rs7176005)	Frequency of block	Frequency ratio % (case:control)	Odds ratio (95%) CI	*P*-value
ACGGC	0.348	0.3189:0.3765	1	N/A
GAGGC	0.2543	0.428 l: 0.2662	1.12 (0.78–1.62)	0.54
GCGGC	0.1914	0.191: 0.1927	1.16 (0.77–1.75)	0.48
ACAGT	0.0818	0.099: 0.0645	1.88 (0.94–3.76)	0.076
GCAGT	0.0612	0.0677: 0.0544	1.69 (0.83–3.47)	0.15

Global haplotype association *p*- value:0.066

*N/A* not applicable

*P*-Value < 0.05 considered as significant

## Discussion

Polymorphisms in the CYP genes have been reported to modulate the risk and development of breast cancer (BC) as well as individual response to anticancer drugs [3, 4]. As previously mentioned, the present study investigated the role of certain *CYP19A1*, *CYP2C9*, *CYP2C19, CYP1B1, CYP3A4,* and *CYP1A2* polymorphisms on BC in Jordanian patients and healthy volunteers.

With regard to *CYP19A1* polymorphisms, rs7176005 and rs6493497 result in variable response to aromatase-inhibitor treatment in early-stage BC patients, while another study found that these polymorphisms did not increase estrogen biosynthesis in postmenopausal women [33, 34]. In a meta-analysis of over 20,000 cases and controls, it was found that rs10046 on its own did not increase BC risk [35]. A systematic review of a dozen studies reported that the *CYP19A1* SNP rs4646 conferred a beneficial effect in that it increased the progression time in patients with metastatic BC [36]. Similarly, the rs700519 SNP of the *CYP19A1* gene was not significantly associated with BC in any ethnic or mixed population in one meta-analysis, but the rs700519 SNP was significantly associated with BC susceptibility in the Han Chinese population [37, 38]. In contrast, our findings show that only the *CYP19A1* SNPs rs7176005 and rs6493497 were significantly associated with BC in the Jordanian population. However, sample selection bias may be encountered in this study. Even though the patients were randomly selected to avoid potential bias, 46% of BC patients suffered from other complications such as hypertension, coronary artery disease, asthma, and diabetes mellitus that might influence the results. On the other hand, we found that the *CYP19A1* SNPs rs7176005 and rs10046 were significantly associated with body mass index and age at BC diagnosis, while rs700519 was linked to both age at menopause and age at menarche.

With regard to *CYP2C9*, the rs1799853 SNP was found to be significantly associated with cyclophosphamide toxicity when analyzed as a part of a haplotype [39]. However, the rs1799853 SNP was not associated with BC in Asian Singaporeans, nor was it associated with increased BC risk in women undergoing menopausal hormone therapy [13, 40]. In our study, none of the investigated *CYP2C9* SNPs were significantly associated with BC or its prognostic factors in Jordanian females. On the other hand, the *CYP2C19* SNP rs4244285 was not associated with BC risk nor with estrogen levels in the German population, but it was associated with longer BC survival rates compared to the wild type [14, 41]. In the Thai population, the rs4244285 SNP was the most common variant but it was not associated with tamoxifen efficiency, while, in the Dutch population, this SNP was significantly linked to time tamoxifen failure [42, 43]. Similar to the *CYP2C9* SNPs, our findings illustrate that the *CYP2C19* SNP rs4244285 is not associated with BC risk or any of its prognostic factors in Jordanians.

In terms of *CYP1A2*, the rs762551 polymorphism was not associated with BC risk or modulated estrogen levels in the German populations, but it resulted in altered BC risk in the Thai population [40, 44, 45]. According to two different meta-analyses, the *CYP1A2* SNP rs762551 was associated with cancer susceptibility in the Caucasian but not the Asian or mixed populations [46, 47]. However, another meta-analysis comprising 17,600 cases and controls found no significant association between rs762551 and BC development in any ethnic population [48]. Our findings show that, in Jordanian BC patients, the *CYP1A2* SNP rs762551 was not associated with BC risk, but it was significantly related to the following BC prognostic factors: age at menopause, histological class, HER2 marker status, and lymph node involvement. This polymorphism was also significantly associated with the heteromolecular marker classes L.A vs L.B. vs T.N.

Concerning the *CYP3A4,* the rs35599367 SNP was significantly associated with exemestane concentration in postmenopausal BC patients, nor was it associated with dose reduction or peripheral neuropathy in paclitaxel-treated BC patients [49, 50]. However, it was associated with higher everolimus concentrations in the blood of postmenopausal BC patients [51]. The findings of the present study show that the *CYP3A4* SNP rs35599367 was not associated with BC risk, but it was significantly associated with age at first pregnancy and tumor stage. Regarding the *CYP1B1*, it has been previously reported that rs10175368 has negligible influence on BC risk in the Caucasian and Polish population [52, 53]. Correspondingly, our findings showed no significant association between the *CYP1B1* SNP rs10175368 and BC risk or BC prognostic factors, but an association was found between this SNP and the different BC heteromolecular classifications.

This study can enrich the scarce literature about breast cancer and its implication in the Arab world and in Jordan particularly. In addition, identifying prognostic factors that can predict the risk of cancer development and progression is in demand in clinical practice. In this study, we hypothesized that the genetic markers that potentially induce breast cancer development and progression are different from those involved in breast cancer subtypes and prognosis. Therefore, determining variants that involved in breast cancer prognosis can help in stratifying patients in clinical trials and lead to characterizing the most effective therapy to provide patients with personalized medicine.

On the other hand, a case-control study with a small sample number could lead to selection bias which was the main limitation in this study. However, other potential limitations were avoided; no bias is caused by population stratification, as the Jordanian Arab population is a relatively homogenous population. Moreover, there were no significant difference between case and control groups in term of basic demographic characteristics. In addition, gender bias was excluded as all the participants limited to females. Finally, genotyping in this study was done using the Sequenom MassARRAY® system, which is one of the most error-free, high throughput, accurate, sensitive, and robust sequencing techniques.

## Conclusions

Conclusively, the findings of the current study suggest that certain polymorphisms of the *CYP19A1* and *CYP1A2* genes are implicated in BC risk and development in Jordanian patients. Furthermore, several CYP genes have been found to be significantly associated with BC prognostic factors, resulting in potentially worsened prognoses for carriers of those polymorphisms. However, this study is an exploratory one that, having identified potential BC susceptibility gene polymorphisms, leaves room for future studies to corroborate these findings using a larger sample size.

## Data Availability

The data sets generated and/or analyzed over the course of the study are not publicly available but are available from the corresponding author on reasonable request.

## Abbreviations

AGRF: Australian Genome Research Facility
BC: Breast Cancer (BC)
CYP: Cytochrome P450 (CYP)
DNA: Deoxyribonucleic acid (DNA)
ER: Estrogen Receptor
HER2: Human epidermal growth factor receptor 2 marker
Het: Heterozygote
HWE: Hardy-Weinberg equilibrium (HWE)
Hz: Homozygote
IRB: Institutional Review Board
JRMS: Jordanian Royal Medical Services
PR: Progesterone Receptor
SNPs: Single nucleotide polymorphisms
SPSS: Statistical Package for the Social Sciences
χ^2^: Chi squared value (χ^2^)
